# Supplementary material for: N-Glycosylation of LRP6 by B3GnT2 Promotes Wnt/β-Catenin Signalling
Source: Cells. 2023 Mar 10;12(6):863. doi: 10.3390/cells12060863 (PMC10047360; doi:10.3390/cells12060863)
Supplement: Supplementary file 1 [file cells-12-00863-s001.zip › cells-2222657-supplementary.pdf]

Supplementary Figure S1

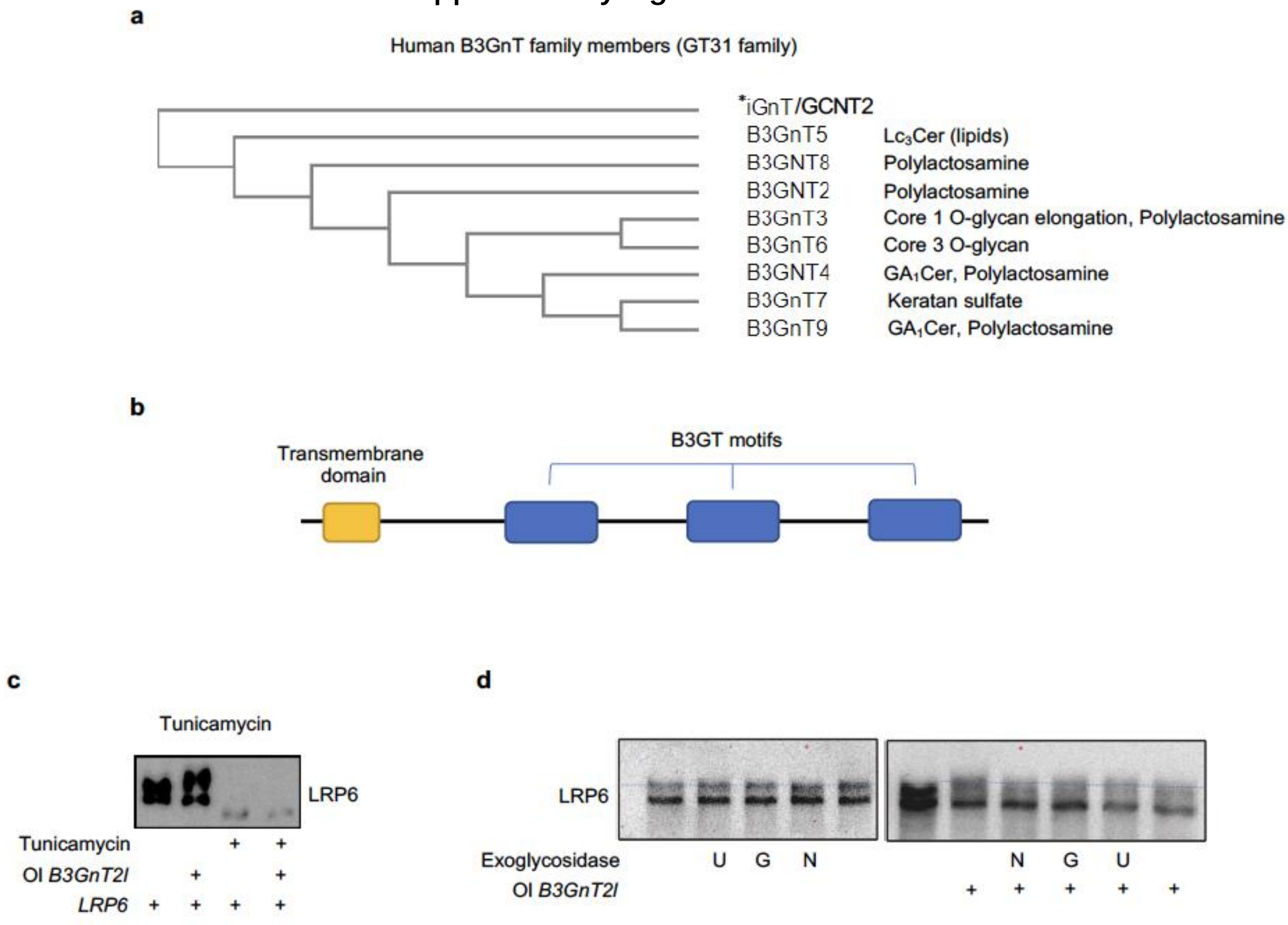

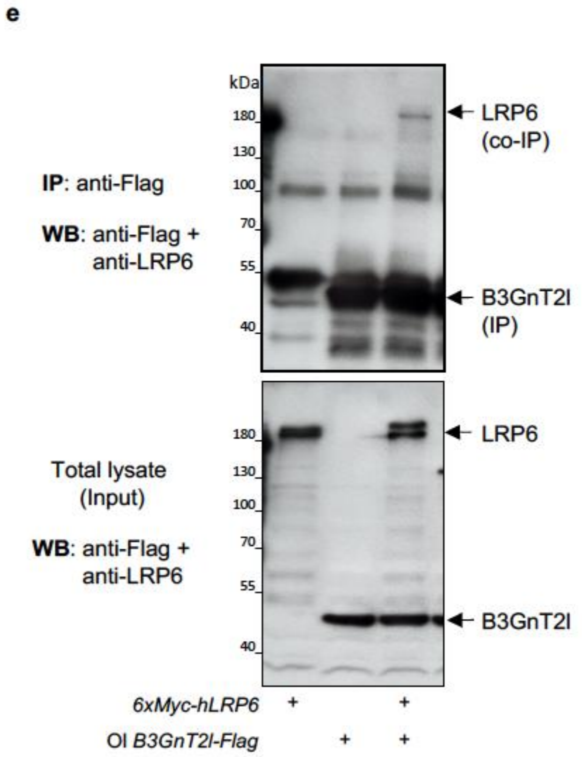

Supplementary Figure S1: (a) Tree diagram of the 8 human B3GnT members within the GT31 family. The guide tree was constructed by Clustal Omega using amino acid sequences. * Note that the I-branching enzyme iGnT/GNT2A used here as a reference is a beta-1,6-N-acetylglucosaminyltransferase. **(b)** B3GnT family members have three conserved B3GT motifs that project into the lumen of the Golgi. **(c)** Western blots of cell lysates from HEK293T cells transfected with the indicated plasmids in 96-well format. 0,1 ug tunicamycin was added 3,5 h after transfection. Amounts transfected: *hLRP6*, 20 ng; *OlFlag-B3GnT2l*, 15ng. **(d)** B3GNT2l modifies the complex N-glycans of LRP6. LRP6 Western Blots of lysates from HEK293T cells in 96-wells transfected with h*LRP6* (20ng) and Ol*B3GNT2l* (5ng) and harvested after 24 hours. Neuraminidase (N), Galactosidase (G), or N-Acetylglucosaminase (U) were added to lysates in Laemmli loading buffer for 2h at 37ºC. Note the small decrease of distance between upper and lower LRP6 bands specifically with Neuraminidase (N). The fine dashed blue line helps to show the subtle difference. **(e)** Western Blots of immunoprecipitates (IP) and their corresponding total cellular lysates from cells transfected in 6-wells as indicated. Anti-Flag-M2-Agarose beads (Sigma-Aldrich) were used to IP B3GnT2l and the upper blot shows that LRP6 proteins were co-immunoprecipitated (co-IP) together with the glycosyltransferase.

Supplementary Figure S2

a

Identity of human B3GnT family members with Ol B3GnT2l

Human B3GnT family member	Identity to Ol B3GnT2-like
B3GnT2	158/462 (34.2%)
B3GnT8	147/439 (33.5%)
B3GnT7	145/450 (32.2%)
B3GnT6	136/446 (30.5%)
B3GnT3	134/469 (28.6%)
B3GnT4	133/480 (27.7%)
B3GnT9	137/498 (27.5%)
B3GnT5	103/497 (20.7%)
iGnT/GCNT2	89/482 (18.5%)

b

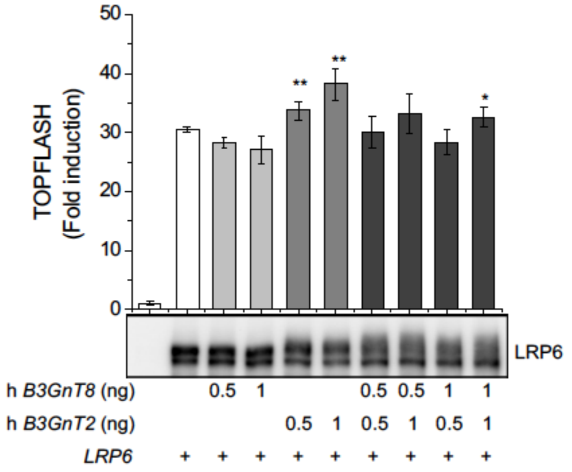

c

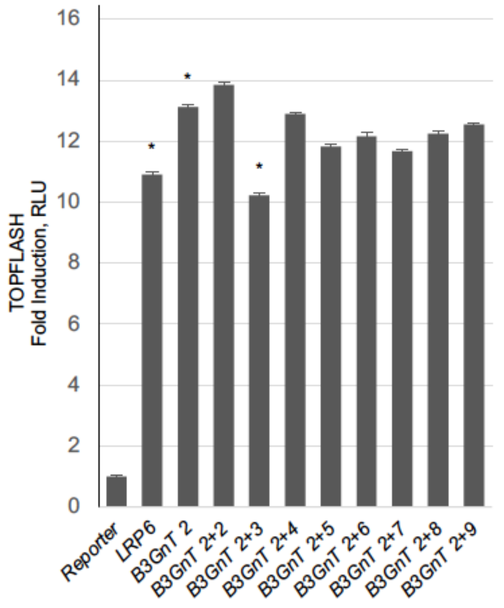

d

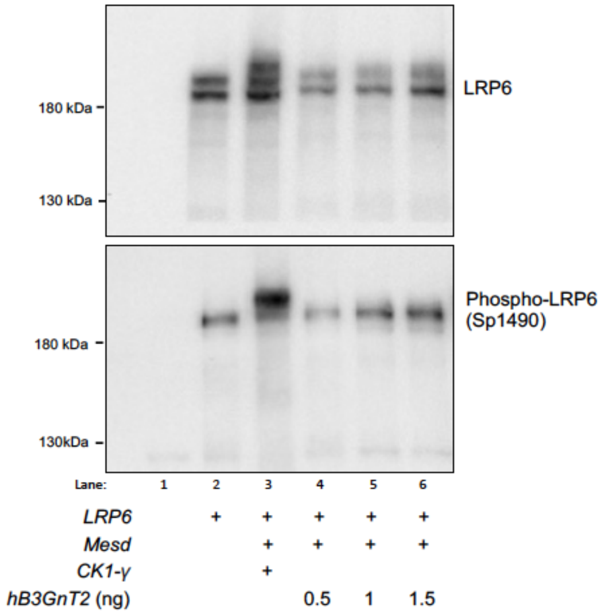

Supplementary Figure S2: (a) Comparison of Ol B3GnT2l identity to human B3GnT family members and iGnT. B3GnT2 and B3GnT8 have the highest sequence identity to medaka B3GnT2l. Alignment was done by EMBOSS Needle using amino acid sequences. **(b).** TOPFLASH Wnt reporter assay (upper graph) and LRP6 Western Blot analysis demonstrating that human B3GnT2 and B3GnT8 do not synergize to promote LRP6/Wnt signaling. Amounts transfected: ***hLRP6***, 20 ng; ***mMesd***, 5 ng; ***hB3GnT2/8***, as indicated. Data represent mean + SD. *P < 0.05; **P < 0.01. (c) TOPFLASH Wnt reporter assay showing effect of ***B3GnT2*** when co-expressed with other ***B3GnT*** family members on LRP6/Wnt signaling. Amounts transfected: ***hLRP6***, 20 ng; ***mMesd***, 5 ng; ***hB3GnT's***, 0,5 ng. Data represent mean + SD. *P < 0.05. **(d)** B3GNT2l does not significantly alter the phosphorylation status of LRP6. Total and phospho- LRP6 Western Blots of lysates from HEK293T cells in 96-wells transfected with *LRP6* (20ng), *Mesd* (5ng), *CK1g* (10ng) and the indicated amounts of ***hB3GnT2*** and harvested after 24 hours. Note the expected upshift of the upper LRP6 bands with B3GnT2 (lanes 4-6), without any detectable increase in the phosphorylation sate (Sp1490 blot). CK1γ was used to promote phosphorylation of LRP6 and resulted in the expected upshift (upper panel) as well as Sp1490 signal (lower panel). Note also the slight reduction of signal in lane 4, which is due to a slightly reduced sample loading in this lane of the gel.

Supplementary Figure S3

a

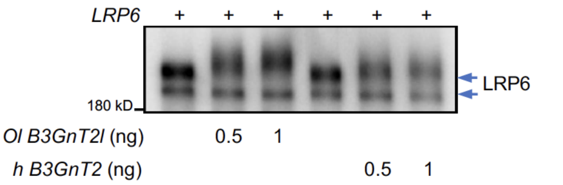

b

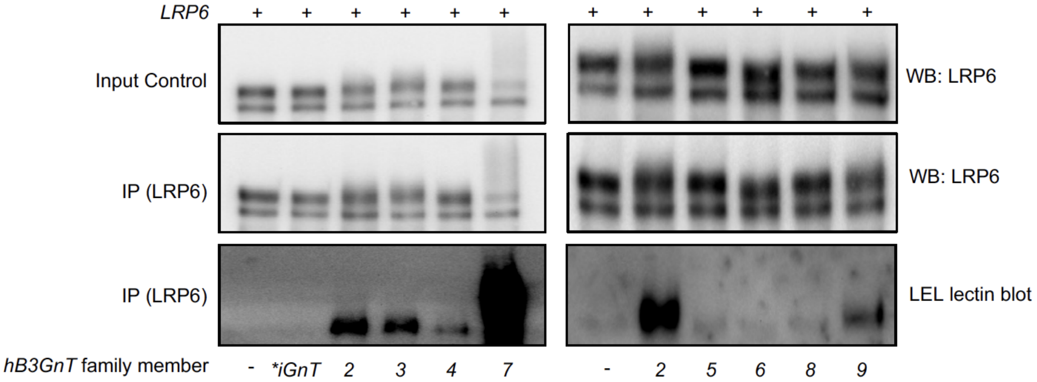

c

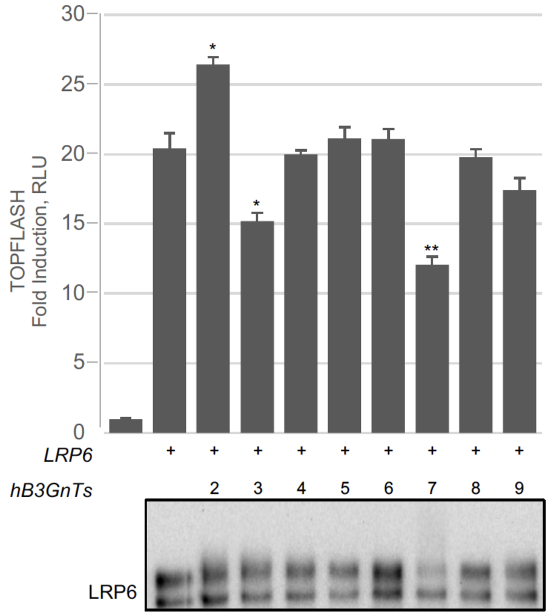

Supplementary Figure S3: (a) Western blots of cell lysates from HEK293T cells transfected with indicated plasmids in 96-well format, showing that Ol B3GnT2l has stronger modification activity than human B3GnT2. Amounts transfected: *hLRP6*, 20 ng; *Ol B3GnT2l*, human *B3GnT2*, as indicated. **(b)** LEL lectin blot analysis of LRP6 using all 8 human B3GnT family members. LRP6 Western blots and lectin blots of cell lysates from HEK293T cells transfected with indicated plasmids in 6-well format. Amounts transfected: *hLRP6*, 600 ng; *mMesd*, 450 ng; *hB3GnTs*, 30 ng. * Note that the I-branching enzyme iGnT/GNT2A used here as a reference control is a beta-1,6-N-acetylglucosaminyltransferase. **(c)** TOPFLASH Wnt reporter assay (top graph) and LRP6 Western Blot (lower panel) showing the effect of all human B3GnT members on LRP6 modification and Wnt/β-catenin signaling. Amounts transfected: *hLRP6*, 20 ng; *mMesd*, 5 ng; *hB3GnTs*, 0.5 ng. **(d)** MS fragmentation spectrum of the N-glycopeptide $[M+3H]^{3+}$ at m/z 1209.4530 (see Figure 3c, N433)

d

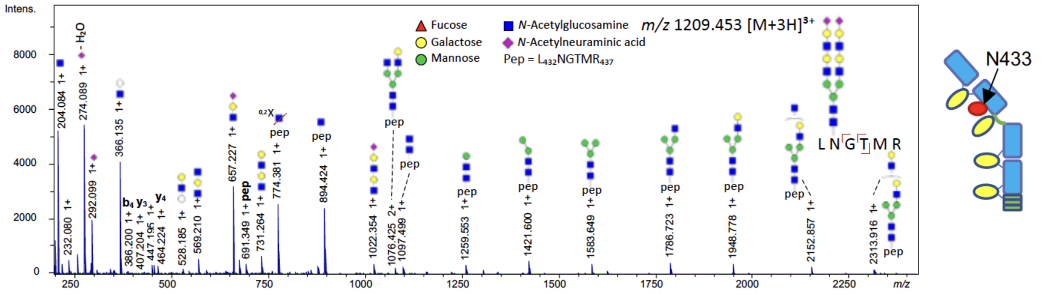

Supplementary Figure S4

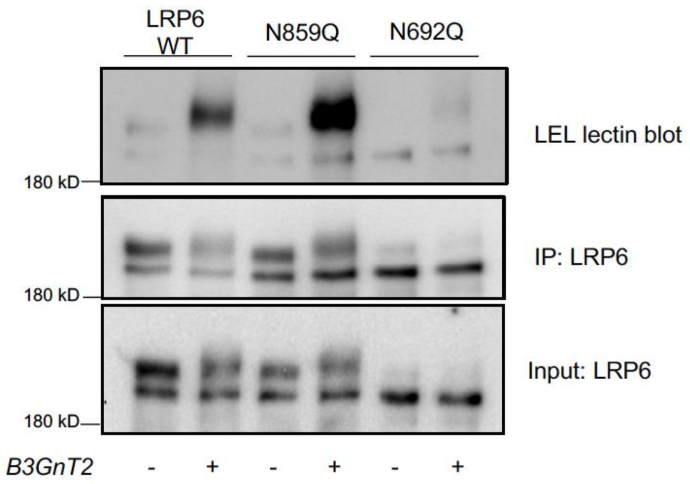

Supplementary Figure S4: LRP6 Glycosylation site Mutant N859Q shows enhanced B3GnT2-mediates polylactosamines modification. Western blot and lectin blots of IP's from HEK293T cells transfected as indicated in 6-well plate. Amounts transfected: indicated *hLRP6*, 600 ng; *mMesd*, 150 ng; *hB3GnT2*, 30 ng.

Supplementary Figure S5

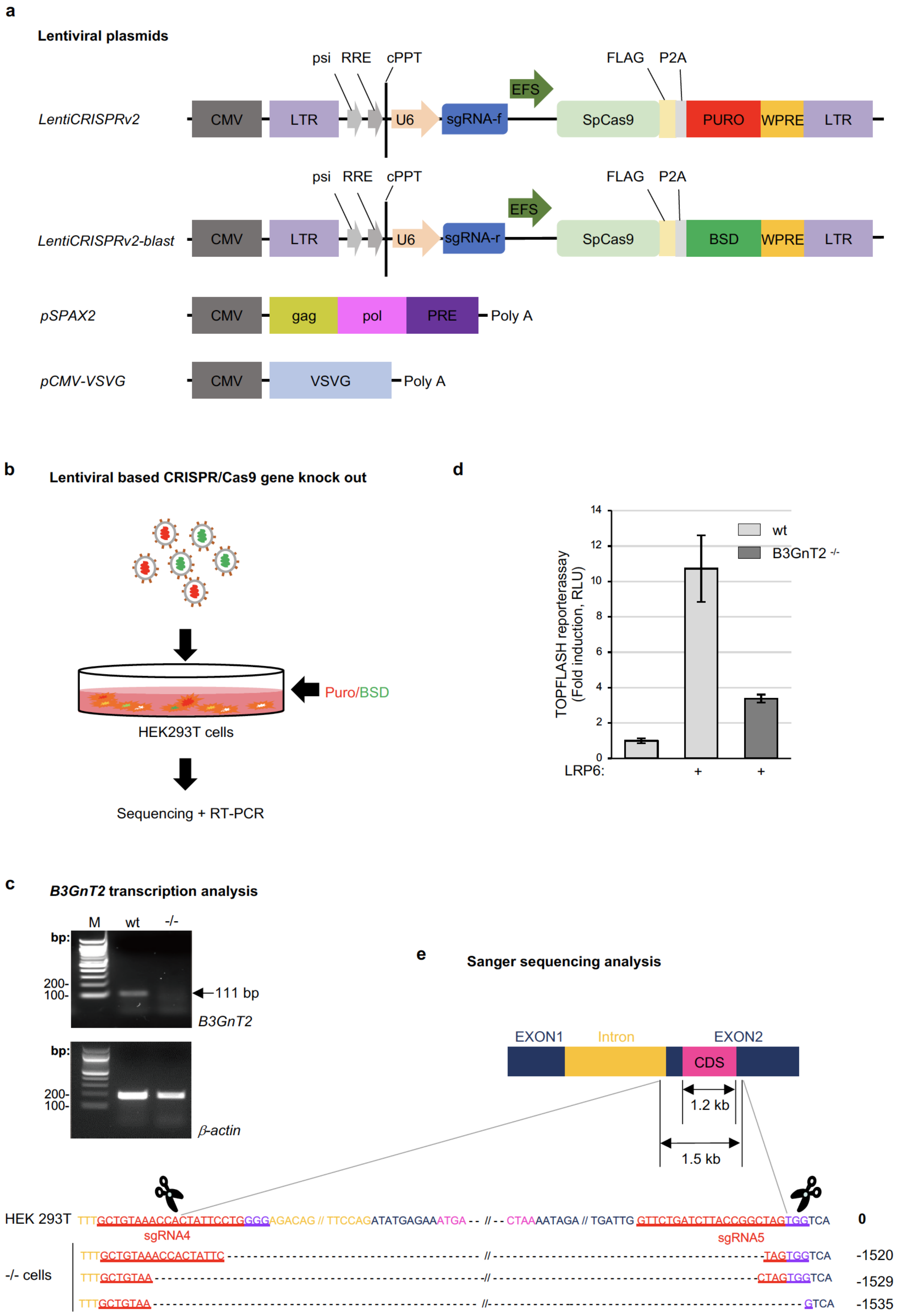

Supplementary Figure S5: lentiviral mediated CRISPR-Cas 9 ablation of human *B3GnT2* gene. **(a)** Lentiviral CRISPR-Cas9 vectors used. CMV, cytomegalovirus immediate early promoter; LTR, long terminal repeat; psi+, HIV-1 packaging signal psi; RRE, HIV-1 response element; cPPT, central polypurine tract; U6, RNA polymerase III promoter; sgRNAF/R, single guide RNA forward/reverse; EFS, elongation factor 1α short promoter; spCas9, Cas9 endonuclease from the Streptococcus pyogenes Type II CRISPR/Cas system; Flag, Flag tag; P2A, 2A self-cleaving peptide; Puro, Puromycin resistance gene; BSD, blastincidin resistance gene; WPRE, Woodchuck hepatitis virus posttranscriptional regulatory element; gag, lentiviral structural protein; pol, precursor protein encoding viral protease, reverse transcriptase and integrase; PRE, HIV-1 rev response element; VSVG, vesicular stomatitis virus G protein gene; Poly A, polyadenylation signal. (**b)** Schematic of Lentiviral based CRISPR/Cas9 gene knock out workflow. HEK 293Tcells were infected with two kinds of lentiviral harbouring the Cas9 and a pair of sgRNAs cutting both end of gene CDS region. The infected cells undergo dual antibiotic selection and quickly checked for the CDS region deletion result. After confirmation of expected deletion in the mixed cell poll, cells were subjected to limited dilution for single colony and genotyping PCR screening steps. The selected gene KO hits were further verified by transcription analysis and Sanger sequencing analysis. (**c)** Transcription analysis for B3GnT2 mRNA expression level on selected hits. B3GnT2 wt allele has a PCR product of 111 bp. **(d)** TOPFLASH reporter assay in HEK293T wild-type and B3GnT2$^{-/-}$ cells transfected with *hLRP6* (20 ng) as indicated. Cells were transfected in 96-wells in tetraplicates and cell lysates harvested 24 h post transfection. All error bars shown are standard deviation (SD) from mean. **(e)** Multiple Sanger sequence alignment of CDS region from B3GnT2 KO clones and HEK 293T wt cells. Gene and sgRNA structure of B3GnT2 were showed in color code. Pink: CDS region; Yellow: Intron sequence; Dark blue: Exon sequence; Red: sgRNA sequence; Purple: PAM sequence.
